# Is Sub-Saharan Africa prepared for COVID-19?

**DOI:** 10.1186/s41182-020-00206-x

**Published:** 2020-04-10

**Authors:** Edwin Nuwagira, Conrad Muzoora

**Affiliations:** Infectious Diseases Unit, Department of Medicine, Mbarara University of Science and Technology, P.O Box 1410, Mbarara, Uganda

**Keywords:** COVID-19, Sub-Saharan Africa

## Abstract

Severe acute respiratory syndrome coronavirus 2 (SARS-CoV-2), the virus that causes coronavirus disease 2019 (COVID-19), is the latest virus to cause global health panic. Due to the rapidly escalating numbers of new infections outside China, COVID-19 was declared a pandemic by the World Health Organization (WHO) on March 11, 2020, in a message delivered by Dr. Tedros Adhanom Ghebreyesus, the WHO Director-General [1]. As of March 21, about 166 countries globally had recoded cases of the COVID-19 in only 4 months since its outbreak in Wuhan, Hubei Province, China [2, 3]. With the Antarctic continent unaffected, Africa, in particular Sub-Saharan Africa (SSA), has recorded the least number of cases, despite the cited moderate-to-high risk of infection [4]. The biggest challenge is whether Sub-Saharan Africa is ready for this pandemic.

To the Editor,

A number of big cities in Asia and Europe have been deserted due to the fear of COVID-19, the most recent emerging infectious viral illness. Apart from the Antarctica, Africa is one of the least affected continents with less than 1000 cases across the continent and only 27 deaths as of March 21, 2020 [2]. This is probably a matter of time since there were until recently frequent and large travel volumes between Asia and Africa due to the good commercial relationships between the two continents and a number of countries in Africa receiving a high number of airline travelers from high-risk cities within China [4]. In fact, over the past decade, air transport between China and Africa has risen to about 630% as a result of the rapid expansion of Chinese investment in Africa [5].

SSA, however, already faces several health challenges such as limited access to intensive care units, for example, in Uganda, there are only 55 intensive care unit (ICU) beds to serve 40 million people, 80% of which are located in the capital, and the nurse to patient ratio of 1:8 making it the worst in the region [6]. High-income countries (HICs) that have been affected by the disease are also facing inadequacy of ICUs. For example, Italy, which has the eighth-highest nominal gross domestic product (GDP) in the world [7], and one of the greatest health systems with approximately 3.2 hospital beds per 1000 people (compared to 2.8 in USA), has found it hard to meet the needs of the overwhelming numbers of critically ill patients, some of whom are specialized health workers [8, 9]. This shows that no one was adequately prepared for the alarming numbers of COVID-19 cases.

Worse still, there is inadequate manpower especially of critical care doctors and consultant anesthetists to run the few ICUs in SSA. There is also a high burden of infectious diseases with emerging viral illnesses such as Ebola hemorrhagic fever and Lassa hemorrhagic fever in addition to the “priority infections” HIV, malaria, and tuberculosis, and for all disease models, mortality has always been the highest in SSA compared to HICs. These infections present with fever, a screening symptom and sign for the COVID-19 making it hard to determine which patients to screen. In the West African Ebola outbreak, fever was used as a screening test, but 10% of the confirmed cases did not have fever, and a number of them are with malaria co-infection [10].

We therefore anticipate human-to-human transmission due to undiagnosed, symptomatic, and asymptomatic patients due to the limited access to personal protective equipment such as N95 masks, respiratory isolation rooms, goggles, and powered air-purifying respirators (PAPR). The application of the prevention measures such as heightened surveillance, rapid identification of cases, patient transfer to isolation, rapid diagnosis, and tracing and follow-up of contacts varies and needs to be improved at all entry points, but this comes at a cost that may not be sustainable. Modeling studies have shown that travel restrictions of up to 90% have a minimal effect on transmission if not coupled with other public health and behavioral change interventions [11]. The travel bans because of COVID-19 will also greatly affect the economy and delay achieving development goals given that most countries in SSA depend on foreign aid [12].

It is therefore important for African governments and WHO to invest immediately in preparedness for the worst-case scenario given that each African country is at a risk of importing COVID-19 from China or other affected countries because of the high volume of air traffic and trade between China and Africa [5]. The WHO, Centers for Disease Control and Prevention in Africa (Africa CDC), and other humanitarian organizations should be ready for an African crisis which we anticipate to be greater than it is in other regions due to its weak health systems. On Thursday, March 19, 2020, the Director-General of WHO, Dr. Tedros, stated that this was an opportunity for Africa to wake up and prepare for the worst [13].

Epidemiologists should provide accurate predictions that will prepare African governments and political leaders with information that will guide them design control measures at different health centers and regional and national levels. Lastly, there is a lot that we can learn from the Ebola success story to save Africa especially by community engagement. The WHO noted the lack of capacity to contain the West Africa Ebola outbreak in 2014, making it difficult to implement infection prevention procedures [14]. We expect the same barriers from the community as noted in the recent Ebola hemorrhagic fever outbreak in the Democratic Republic of Congo (DRC), such as mistrust of response teams, beliefs that the virus was fabricated, and non-compliant intentions such as hiding from health authorities with discourse [15]. Behaviors like hand shaking, social gatherings, breach of quarantine rules, and contact with the dead may see the number of cases increase in Sub-Saharan Africa.

## Data Availability

All the data used in this in this letter are drawn from the references provided.

## Abbreviations

CDC: Centers for Disease Control and Prevention
COVID-19: Coronavirus disease 2019
DRC: Democratic Republic of Congo
GDP: Gross domestic product
PAPR: Powered air-purifying respirators
SSA: Sub-Saharan Africa
WHO: World Health Organization
